# Supplementary figures and images for: How multisite phosphorylation impacts the conformations of intrinsically disordered proteins
Source: PLoS Comput Biol. 2021 May 4;17(5):e1008939. doi: 10.1371/journal.pcbi.1008939 (PMC8148376; doi:10.1371/journal.pcbi.1008939)

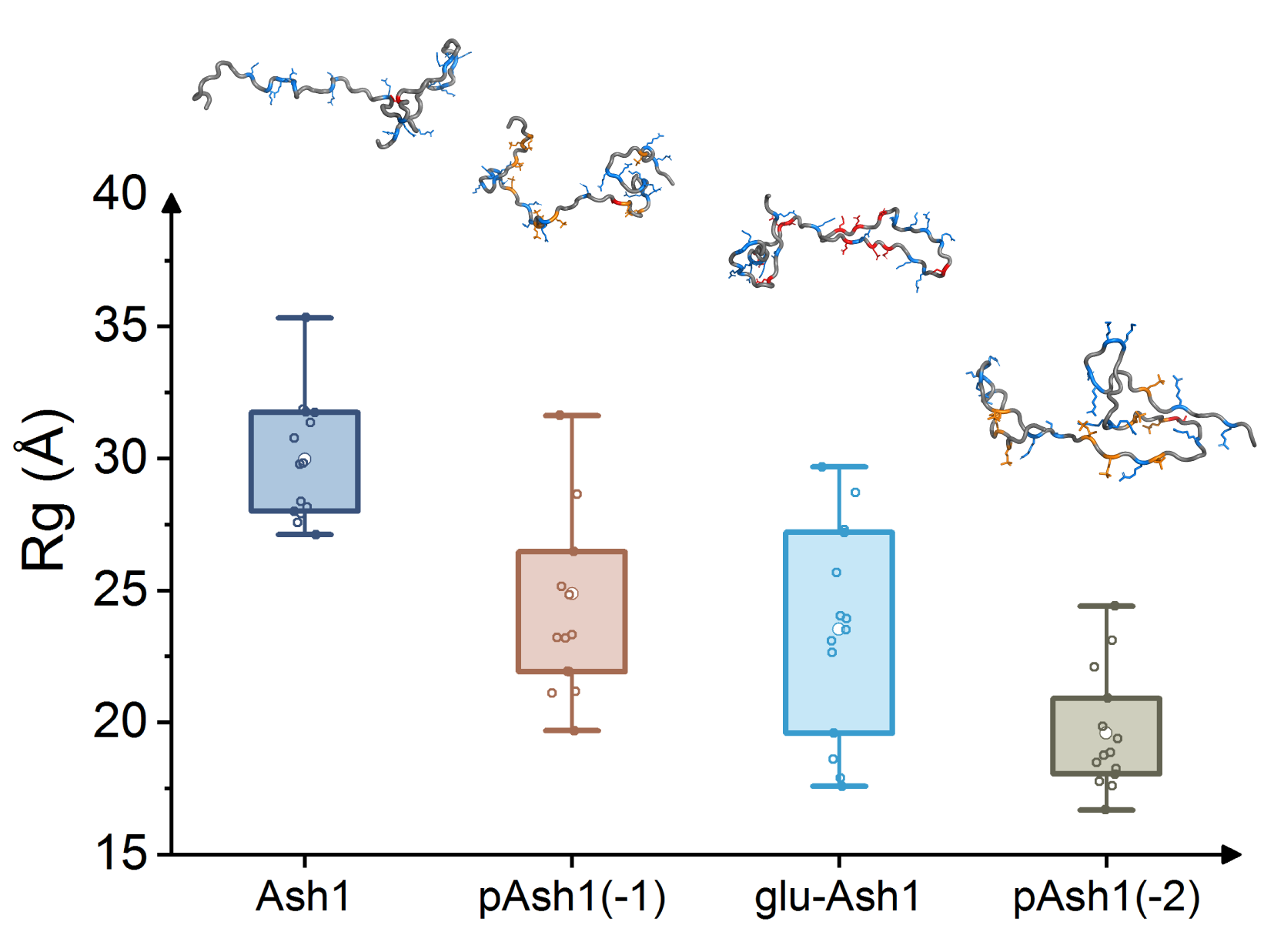

Supplement: S1 Fig — (TIF) [file pcbi.1008939.s001.tif]

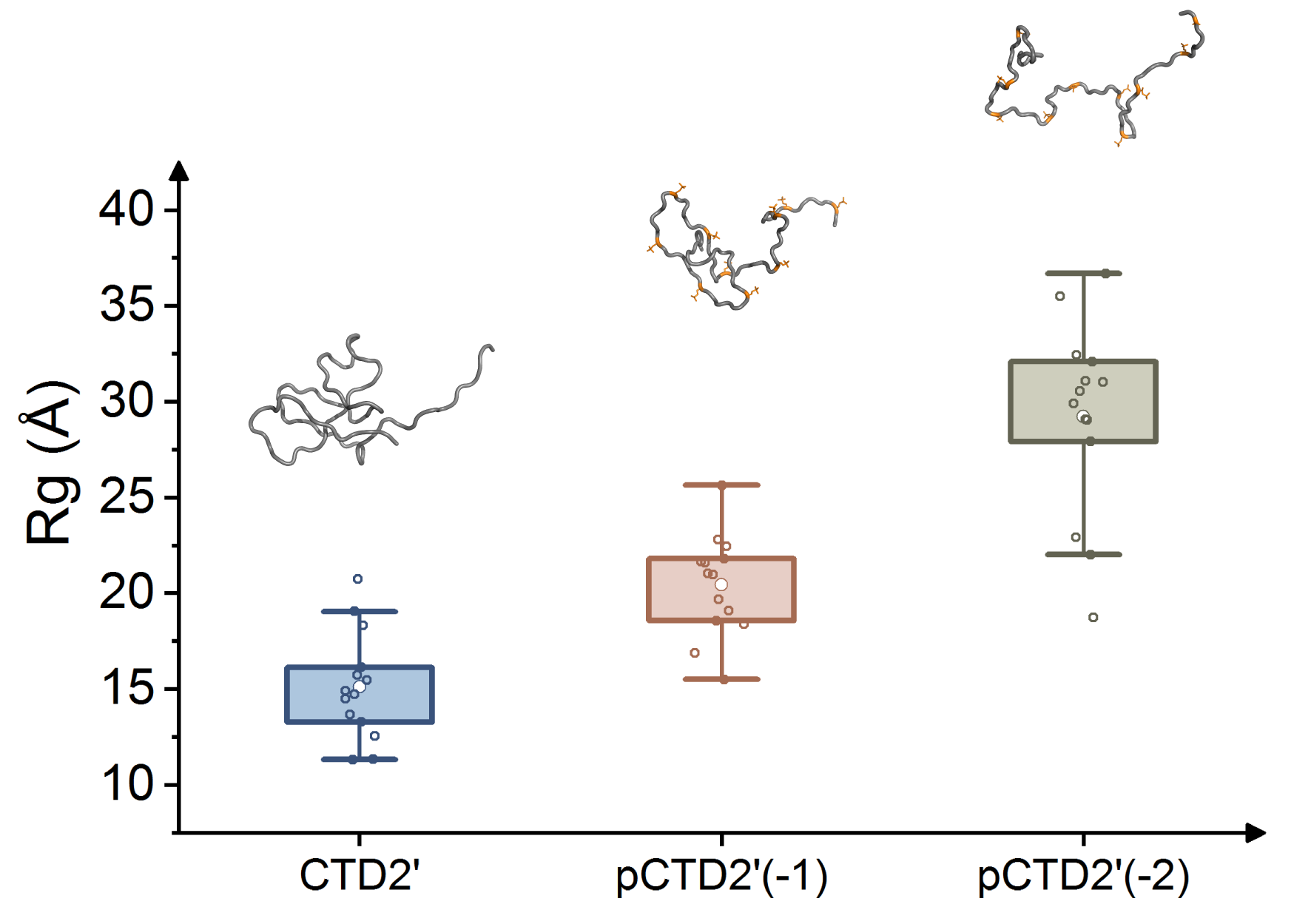

Supplement: S4 Fig — Global conformational changes in 100 mM NaCl, measured by RG, the mass-weighted radius of gyration, of CTD2’ for unphosphorylated (blue) and multi-site phosphorylated forms (HPO4-1 in red and PO4-2 in olive). (TIF) [file pcbi.1008939.s004.tif]

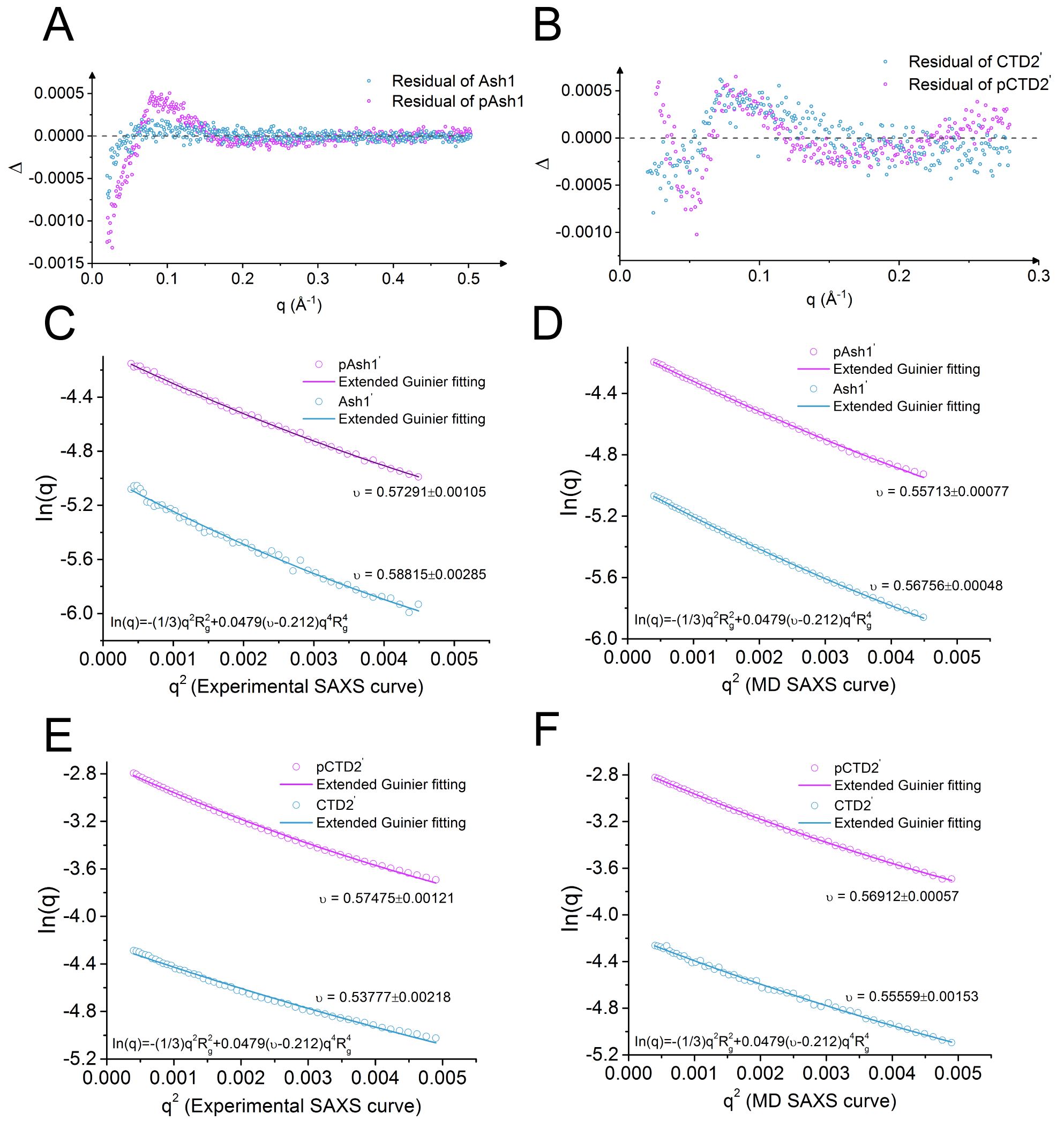

Supplement: S5 Fig — Comparison of MD simulations to SAXS data. Residuals of fitting for Ash1 (A) and CTD2’ (B) Δ = [Icalc (q) —Iexp (q)], q is the scattering vector. Guinier analyses were conducted for Ash1 (C and D) and CTD2’ (E and F) for experimental and MD back-calculated SAXS curve. (TIF) [file pcbi.1008939.s005.tif]

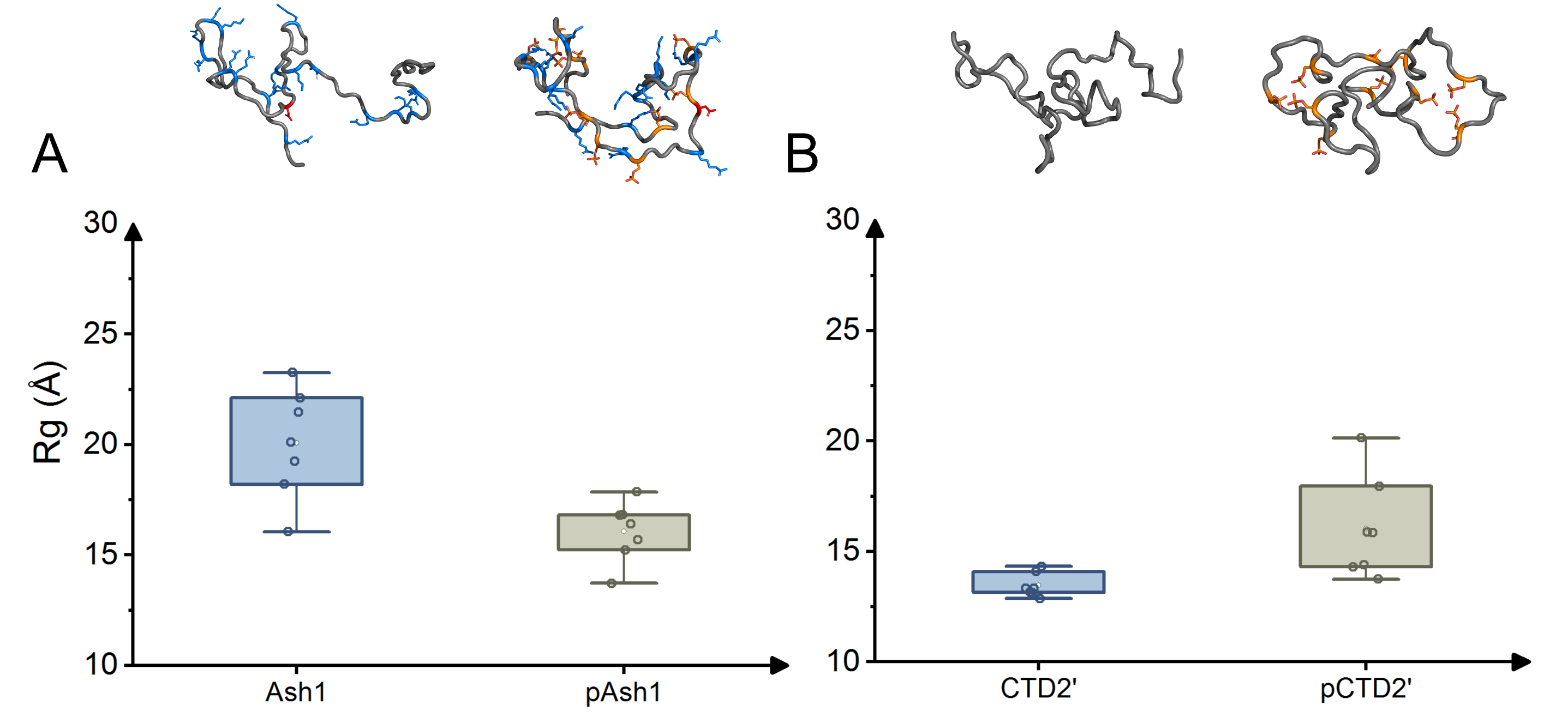

Supplement: S6 Fig — Global conformational changes in 100 mM NaCl, measured by RG, the mass-weighted radius of gyration, of Ash1 (A) and CTD2’ (B) for unphosphorylated (blue) and multi-site phosphorylated forms (PO4-2 in olive) by using the Charmm36m forcefield. The central structures of each state are shown in cartoon, with positively charged and negatively charged residues shown in blue and red, respectively. The phosphorylated residues are shown as orange sticks. (TIF) [file pcbi.1008939.s006.tif]

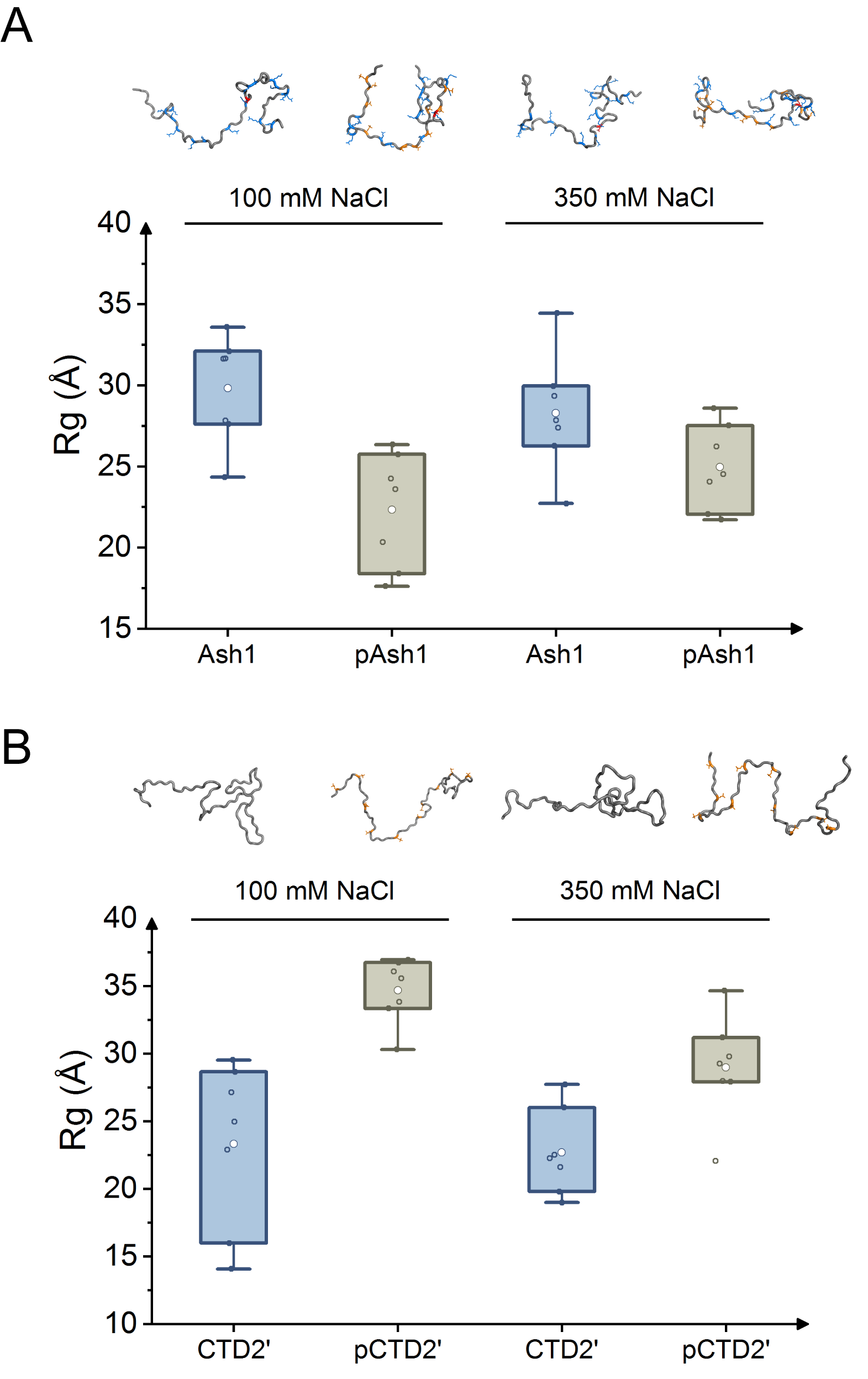

Supplement: S7 Fig — Global conformational changes in 100 and 350 mM NaCl, measured by RG, the mass-weighted radius of gyration, of Ash1 (A) and CTD2’ (B) for unphosphorylated (blue) and multi-site phosphorylated forms (PO4-2 in olive) when using the a99SB-disp forcefield. (TIF) [file pcbi.1008939.s007.tif]

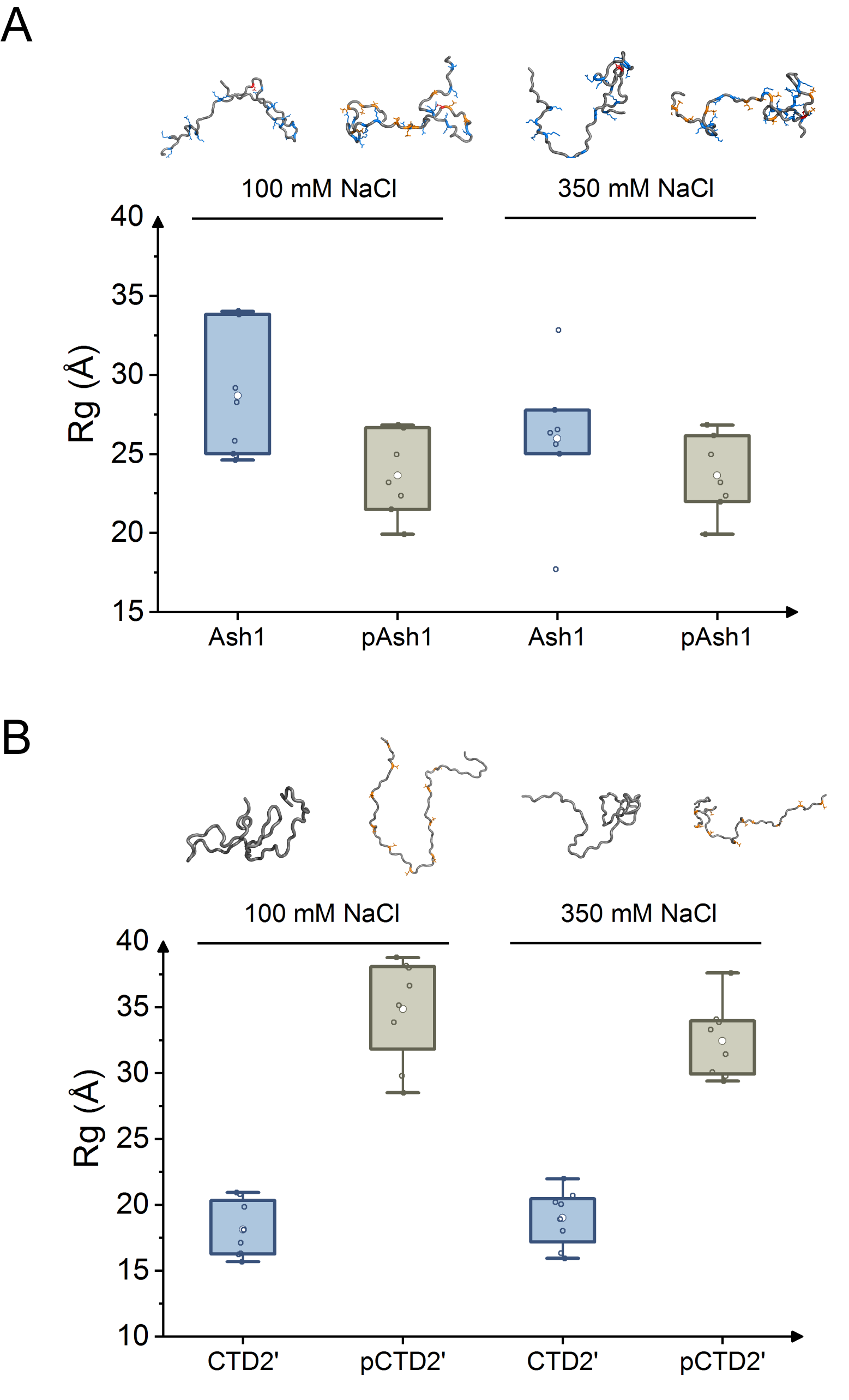

Supplement: S8 Fig — Global conformational changes in 100 and 350 mM NaCl, measured by RG, the mass-weighted radius of gyration, of Ash1 (A) and CTD2’ (B) for unphosphorylated (blue) and multi-site phosphorylated forms (PO4-2 in olive) when using the Amber99SB* ILDN-DERK forcefield. (TIF) [file pcbi.1008939.s008.tif]

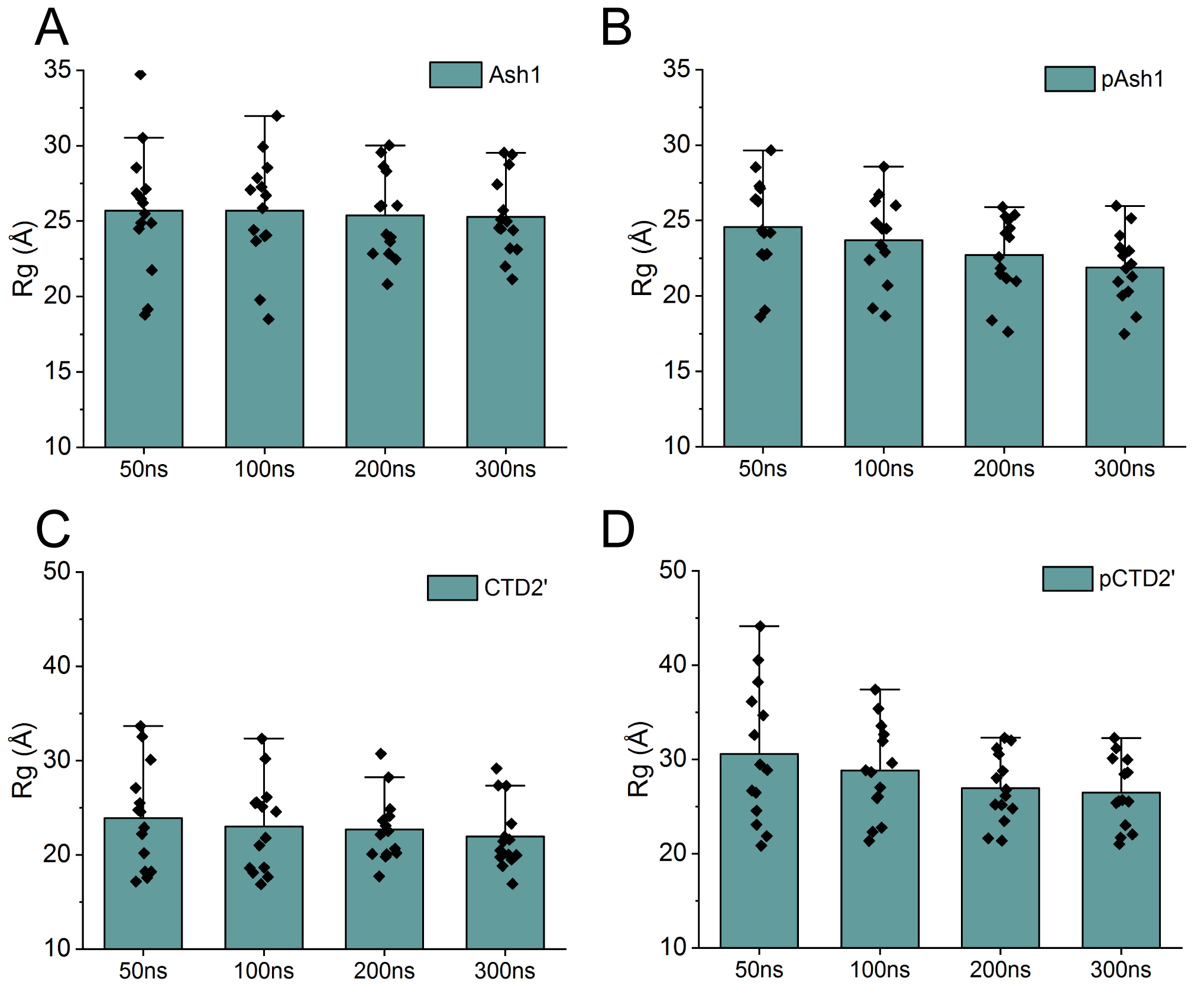

Supplement: S11 Fig — The radius of gyration were compared for MD simulations of Ash1/pAsh1 and CTD2’/pCTD2’ under 350 mM NaCl of 50 ns, 100 ns, 200 ns and 300 ns simulation time. The first 100 ns simulations were regarded as equilibration and not used in analyses. (TIF) [file pcbi.1008939.s011.tif]
