# Supplementary material for: How multisite phosphorylation impacts the conformations of intrinsically disordered proteins
Source: PLoS Comput Biol. 2021 May 4;17(5):e1008939. doi: 10.1371/journal.pcbi.1008939 (PMC8148376; doi:10.1371/journal.pcbi.1008939)
Supplement: S1 Table — (PDF) [file pcbi.1008939.s012.pdf]

350 mM	Ash1	pAsh1
H	0.15	0.17
Cα	0.37	0.54
Cβ	0.73	0.73
C	0.70	0.90
N	1.60	2.26
Average	0.71	0.92
150 mM	Ash1	pAsh1
H	0.16	0.21
Cα	0.40	0.70
Cβ	0.69	0.83
C	0.70	0.94
N	1.93	2.14
Average	0.77	0.96
200 mM	Ash1	pAsh1
H	0.16	0.23
Cα	0.40	0.62
Cβ	0.70	0.75
C	0.72	1.02
N	1.97	2.05
Average	0.79	0.93
250 mM	Ash1	pAsh1
H	0.16	0.21
Cα	0.47	0.86
Cβ	0.83	2.40
C	0.72	1.10
N	2.1	2.19
Average	0.86	1.35
300 mM	Ash1	pAsh1
H	0.15	0.19
Cα	0.36	0.70
Cβ	0.71	0.85
C	0.70	1.02
N	1.86	2.17
Average	0.75	0.98
400 mM	Ash1	pAsh1
H	0.15	0.20
Cα	0.45	0.65
Cβ	0.71	0.76
C	0.70	1.02
N	2.08	2.17
Average	0.81	0.96
500 mM	Ash1	pAsh1
H	0.17	0.18
Cα	0.45	0.64
Cβ	0.74	0.82
C	0.72	0.98
N	1.94	2.08

Average	0.8	0.94
600 mM	Ash1	pAsh1
H	0.15	0.22
Cα	0.44	0.64
Cβ	0.72	0.77
C	0.72	1.00
N	2.17	2.16
Average	0.84	0.96
700 mM	Ash1	pAsh1
H	0.15	0.19
Cα	0.41	0.65
Cβ	0.70	0.76
C	0.71	1.00
N	2.25	2.16
Average	0.84	0.95
800 mM	Ash1	pAsh1
H	0.14	0.21
Cα	0.37	0.61
Cβ	0.77	0.77
C	0.71	1.00
N	2.10	2.14
Average	0.81	0.94
1000 mM	Ash1	pAsh1
H	0.15	0.19
Cα	0.41	0.59
Cβ	0.71	0.76
C	0.74	0.99
N	2.04	2.02
Average	0.81	0.91
1500 mM	Ash1	pAsh1
H	0.15	0.20
Cα	0.37	0.65
Cβ	0.70	0.85
C	0.71	1.00
N	1.94	2.14
Average	0.77	0.97
