# Supplementary material for: How multisite phosphorylation impacts the conformations of intrinsically disordered proteins
Source: PLoS Comput Biol. 2021 May 4;17(5):e1008939. doi: 10.1371/journal.pcbi.1008939 (PMC8148376; doi:10.1371/journal.pcbi.1008939)
Supplement: S2 Table — (PDF) [file pcbi.1008939.s013.pdf]

Step1: Initial structure preparation

IDP	forcefield	Salt concentration	Number of atoms	Simulation time (ns)
Ash1	AMBER99-sb*-ILDN	100, 150, 200, 300, 350, 400, 500, 800, 1000, 1200 mM	Ca. 280,000	1 x 420 (for each salt concentration)
pAsh1[*]	AMBER99-sb*-ILDN	100, 150, 200, 300, 350, 400, 500, 800, 1000 and 1200 mM	Ca. 220,000	1 x 420 (for each salt concentration)
Ash1	AMBER99-sb*-ILDN-DERK	100 and 350 mM	Ca. 250,000	1 x 420 (for each salt concentration)
pAsh1	AMBER99-sb*-ILDN-DERK	100 and 350 mM	Ca. 250,000	1 x 420 (for each salt concentration)
Ash1	A99SB-disp	100 and 350 mM	Ca. 250,000	1 x 420 (for each salt concentration)
pAsh1	A99SB-disp	100 and 350 mM	Ca. 250,000	1 x 420 (for each salt concentration)
CTD2'	AMBER99-sb*-ILDN	100, 150, 200, 300, 350, 400, 500, 800, 1000, 1200 mM	Ca. 200,000	1 x 420 (for each salt concentration)
pCTD2'	AMBER99-sb*-ILDN	100, 150, 200, 300, 350, 400, 500, 800, 1000 and 1200 mM	Ca. 230,000	1 x 420 (for each salt concentration)
CTD2'	A99SB-disp	100 and 350 mM	Ca. 230,000	1 x 420 (for each salt concentration)
pCTD2'	A99SB-disp	100 and 350 mM	Ca. 400,000	1 x 420 (for each salt concentration)
E-Cadherin	AMBER99-sb*-ILDN	100 and 350 mM	Ca. 290,000	1 x 420 (for each salt concentration)
pE-Cadherin (2 P-sites)	AMBER99-sb*-ILDN	100 and 350 mM	Ca. 290,000	1 x 420 (for each salt concentration)
pE-Cadherin (8 P-sites)	AMBER99-sb*-ILDN	100 and 350 mM	Ca. 340,000	1 x 420 (for each salt concentration)
p130Cas	AMBER99-sb*-ILDN	100 and 350 mM	Ca. 290,000	1 x 420 (for each salt concentration)
pp130Cas (2 P-sites)	AMBER99-sb*-ILDN	100 and 350 mM	Ca. 280,000	1 x 420 (for each salt concentration)
pp130Cas (6 P-sites)	AMBER99-sb*-ILDN	100 and 350 mM	Ca. 330,000	1 x 420 (for each salt concentration)
pAsh1 (HPO_4^-)	AMBER99-sb*-ILDN	100 mM	Ca. 280,000	1 x 420
eAsh1[#]	AMBER99-sb*-ILDN	100 mM	Ca. 280,000	1 x 420
pCTD2' (HPO_4^-)	AMBER99-sb*-ILDN	100 mM	Ca. 230,000	1 x 420
pE-Cadherin (-HPO_4^-) (2 P-sites)	AMBER99-sb*-ILDN	100 mM	Ca. 290,000	1 x 420
pE-Cadherin (-HPO_4^-) (8 P-sites)	AMBER99-sb*-ILDN	100 mM	Ca. 340,000	1 x 420

pp130Cas (-HPO_4^-) (2 P-sites)	AMBER99-sb*-ILDN	100 mM	Ca. 290,000	1 x 420
pp130Cas (-HPO_4^-) (6 P-sites)	AMBER99-sb*-ILDN	100 mM	Ca. 340,000	1 x 420
Ash1	Charmm36m	100 mM	Ca. 280,000	1 x 420
pAsh1	Charmm36m	100 mM	Ca. 250,000	1 x 420
CTD2'	Charmm36m	100 mM	Ca. 290,000	1 x 420
pCTD2'	Charmm36m	100 mM	Ca. 280,000	1 x 420

Step 2: Sampling from different initial structure generated from step 1

IDP	forcefield	Salt concentration	Number of atoms	Simulation time (ns)
Ash1	AMBER99-sb*-ILDN	150, 200, 300, 400, 500, 800, 1000, 1200 mM	Ca. 280,000	3 x 400 (for each salt concentration)
pAsh1	AMBER99-sb*-ILDN	150, 200, 300, 400, 500, 800, 1000 and 1200 mM	Ca. 220,000	3 x 400 (for each salt concentration)
Ash1	AMBER99-sb*-ILDN	100 and 350 mM	Ca. 850,000	14 x 400 (for each salt concentration)
pAsh1	AMBER99-sb*-ILDN	100 and 350 mM	Ca. 430,000	14 x 400 (for each salt concentration)
Ash1	AMBER99-sb*-ILDN-DERK	100 and 350 mM	Ca. 250,000	7 x 400 (for each salt concentration)
pAsh1	AMBER99-sb*-ILDN-DERK	100 and 350 mM	Ca. 250,000	7 x 400 (for each salt concentration)
Ash1	A99SB-disp	100 and 350 mM	Ca. 250,000	7 x 400 (for each salt concentration)
pAsh1	A99SB-disp	100 and 350 mM	Ca. 250,000	7 x 400 (for each salt concentration)
CTD2'	AMBER99-sb*-ILDN	150, 200, 300, 400, 500, 800, 1000, 1200 mM	Ca. 200,000	3 x 400 (for each salt concentration)
pCTD2'	AMBER99-sb*-ILDN	150, 200, 300, 400, 500, 800, 1000 and 1200 mM	Ca. 230,000	3 x 400 (for each salt concentration)
CTD2'	AMBER99-sb*-ILDN	100 and 350 mM	Ca. 390,000	14 x 400 (for each salt concentration)
pCTD2'	AMBER99-sb*-ILDN	100 and 350 mM	Ca. 850,000	14 x 400 (for each salt concentration)
CTD2'	A99SB-disp	100 and 350 mM	Ca. 230,000	7 x 400 (for each salt concentration)

pCTD2’	A99SB-disp	100 and 350 mM	Ca. 400,000	7 x 400 (for each salt concentration)
E-Cadherin	AMBER99-sb*-ILDN	100 and 350 mM	Ca. 290,000	14 x 400 (for each salt concentration)
pE-Cadherin (2 P-sites)	AMBER99-sb*-ILDN	100 and 350 mM	Ca. 290,000	14 x 400 (for each salt concentration)
pE-Cadherin (8 P-sites)	AMBER99-sb*-ILDN	100 and 350 mM	Ca. 340,000	14 x 400 (for each salt concentration)
p130Cas	AMBER99-sb*-ILDN	100 and 350 mM	Ca. 290,000	14 x 400 (for each salt concentration)
pp130Cas (2 P-sites)	AMBER99-sb*-ILDN	100 and 350 mM	Ca. 280,000	14 x 400 (for each salt concentration)
pp130Cas (6 P-sites)	AMBER99-sb*-ILDN	100 and 350 mM	Ca. 330,000	14 x 400 (for each salt concentration)
pAsh1 (HPO_4^-)	AMBER99-sb*-ILDN	100 mM	Ca. 280,000	14 x 400
eAsh1	AMBER99-sb*-ILDN	100 mM	Ca. 280,000	14 x 400
pCTD2’ (HPO_4^-)	AMBER99-sb*-ILDN	100 mM	Ca. 230,000	14 x 420
pE-Cadherin (-HPO_4^-) (2 P-sites)	AMBER99-sb*-ILDN	100 mM	Ca. 290,000	14 x 400
pE-Cadherin (-HPO_4^-) (8 P-sites)	AMBER99-sb*-ILDN	100 mM	Ca. 340,000	14 x 400
pp130Cas (-HPO_4^-) (2 P-sites)	AMBER99-sb*-ILDN	100 mM	Ca. 290,000	14 x 400
pp130Cas (-HPO_4^-) (6 P-sites)	AMBER99-sb*-ILDN	100 mM	Ca. 340,000	14 x 400
Ash1	Charmm36m	100 mM	Ca. 280,000	7 x 400
pAsh1	Charmm36m	100 mM	Ca. 250,000	7 x 400
CTD2’	Charmm36m	100 mM	Ca. 290,000	7 x 400
pCTD2’	Charmm36m	100 mM	Ca. 280,000	7 x 400
WAXSIS: Explicit solvent SAXS curve calculation				
Pure water	AMBER99-sb*-ILDN	350 mM	Ca. 1,040,000	200

*: pAsh1, pCTD2’, pE-cadherin and pP130Cas referred to deprotonated form of phosphate group (-PO_4^{2-}) with -2 charge, if not specified.

#: eAsh1 refers to the phosphomimetic version of Ash1 with all phosphorylation sites replaced by Glu.
