# Supplementary material for: How multisite phosphorylation impacts the conformations of intrinsically disordered proteins
Source: PLoS Comput Biol. 2021 May 4;17(5):e1008939. doi: 10.1371/journal.pcbi.1008939 (PMC8148376; doi:10.1371/journal.pcbi.1008939)
Supplement: Attachment — Submitted filename: ResponseToReviewer.docx [file pcbi.1008939.s015.docx]

Point-by-point reply

Reviewer #1: Very timely problem, addressed in a very rigorous manner. Owing to the relevance of the phenomenon studied and the number of not sufficiently careful computational studies on the field, the reviewer is grateful to the authors that they 'pour clean water into the glass'.

We thank you very much for the positive evaluation and the constructive suggestions to improve our manuscript.

Minor comments:
(1) - For general audience, it would be useful to prepare a scheme summarising the effect of phosphorylation in disordered or partly structured domains based on your findings and previous results of others. Can you elaborate the potential mechanisms in the intro, as well?

We have inserted a simple scheme as new Figure 1 to illustrate our hypothesis on IDP phosphorylation. We now discuss this from line 63 of page 4 as below:

“Under the simple hypothesis that electrostatics dominate, phosphorylation of overall negatively charged IDPs is supposed to result in more extended conformations on average while phosphorylation of positively charged IDPs is supposed to result in more compacted conformations. In this view, the dominant driving forces causing a shift of the IDP conformational ensemble across its characteristic flat energy landscape are repulsive/attractive electrostatic forces between the negatively/positively charged residues and the phosphorylated residues (Figure 1).”

(2) - The impact of phosphorylation also depends on to what extent the ID chains get ordered upon binding. Can you elaborate this aspect, in particular in the lights of how phosphorylation can screen protein-protein interactions (autoinhibitory effects)?
I can give a long list of citations of experimental studies eg. on Max, Ets-1, Ire-1, FACT etc., summarised in reviews on fuzzy complexes.

Phosphorylation is very critical for IDP interactions including folding-upon binding, and this is very much related to our defense-attack perspective. We should note that our study is based on only the disordered ensemble, and predictions for transition to the ordered state cannot be (straightforwardly) made. We now cite references 36 and 37 and discuss this point from line 291 of page 14 as below:

“This is particularly important in light of the formation of “fuzzy complexes” [36]. Phosphorylation has in these cases been shown to inhibit or enhance transient interactions of the complex, for example by masking positively charged residues or by inducing cation-π interactions between aromatic and phosphorylated residues [37].”

(3) - I appreciate the large-scale study on different FFs. Can you detail the polarisation effect, solvent models and maybe sampling factors?

Our MD simulations failed to consider polarization effects, but polarizable forcefields are coming of age and could become an interesting choice given an increase in computing power in the future. Here, the solvent models used, e.g. tip4pd, increase the protein-solvent interaction, which is a correction to the overestimation of residue-residue interactions in canonical force fields. For the sampling factor, we consider our sampling very extensive, with differences between force fields showing up already on time scales lower than the simulated time scale. To quantify this, we now have calculated the Rg of Ash1/CTD2’ under 350 mM NaCl for different simulation times (Fig.S10), Simulations with 300 to 400 ns of multiple trajectories are very close to convergence.

We discuss these points from line 372 of page 17 as below:

“Standard force fields are prone to over-stabilize salt bridges [24] and could not deal with polarization of atoms [47]. Here, by increasing the concentration of NaCl (see Results) and using Tip4p-D [19] solvent model, which strengthens the protein-solvent interactions, we partly overcame the limitations of standard protein force fields.

To get an estimate for the convergence of Rg, we analyze the Rg of Ash1 and CTD2’ in their unphosphorylated and phosphorylated forms for different MD times, and observe reasonable convergence within 300 ns to 400 ns (Fig. S11).”

We agree that polarizable ff has a strong potential, referred to them already in the original submission (page 15 line 320, now in blue):

“A higher salt concentration to obtain reasonable conformational ensembles is not a perfect solution for the overstabilization of salt bridges, and future work is required to solve this problem by other parametrizations (e.g. polarizable IDP forcefields).”

(4) - Application to biomolecular condensates. It is good that you mention this, but would be worth to mentioning in intro. Not only dynamic condensates but formation of many different kinds of higher-order assemblies are regulated by phosphorylation (see Wu& Fuxreiter Cell 2016). The question is then, how the screening effect of phosphorylation which you describe relates to the interactions in 'traditional' assemblies and protein droplets. This is a crucial point to allocate the sites of regulation for biomolecular condensates.

We have discussed this point in more detail from line 59 of page 4 as below and cited Wu& Fuxreiter Cell 2016 as reference [5]:

“Furthermore, IDPs are heavily involved in formation of various kinds of higher-order assemblies, and it is crucial to uncover major driving forces of phosphorylation-based regulation of biomolecular condensates [5]”

Reviewer #2: In their manuscript “How multisite phosphorylation impacts the conformations of intrinsically disordered proteins”, Jin and Gräter describe a systematic study in which they use extensive molecular dynamics simulations to characterize the effects of phosphorylation on the structure of IDPs of different amino acid composition. From Table 2 of the SI (list of simulations), it is clear that their study has covered a variety of sequences (Ash1, E-Cadherin, CTD2’ and p130Cas), phosphorylation states, salt concentrations and force fields, for a total sampling time of 0.272 ms. The manuscript is clear and understandable, and the authors have carried out a careful study on an important and current problem in the field of IDPs.
I have some suggestions for revision that could strengthen the manuscript and some questions about details in the manuscript:

We thank you very much for the positive response and valuable feedback.

- In the introduction, it would be relevant to mention recent work from the Skepö lab (<https://pubs.acs.org/doi/abs/10.1021/acs.jctc.9b01190>) on the effect of phosphorylation on the conformation of another IDP - statherin. This work looks at a similar problem as this manuscript.

This article has been published very recently and is closely related to our work, thanks for pointing us to this study. It actually confirms our case: salt bridges appear to overcompact the phosphorylated state of statherin. We now discuss it from line 89 of page 5 as below and cite it as reference [29]:

“More recently, the conformational changes upon phosphorylation of a short IDP fragment of statherin were investigated by comparing the MD simulations using AMBER ff99SB-ILDN and CHARMM36m force fields with SAXS and circular dichroism experiments [29]. The two force fields performed less well for the phosphorylated than for the unphosphorylated states of statherin and also showed differences between each other with regard to secondary structure propensities.”

And again on page 9 lines 167:

“Our finding is also in line with the observation of an over-compacted phosphorylated state of statherin stabilized by a salt bridge [29]. “

- Table 1 — it would be helpful to provide the Genbank accession numbers for the sequences because it is unclear what species they are from.
We have provided the UniProt ID in Table 1. The UniProt IDs are shown below the name of the IDPs.

- On p. 8 line 145, the authors cite ref. 25 for the statement “Given that the current protein force fields have been shown to overstabilize electrostatic interactions“, but as far as I can tell, reference 25 doesn’t support that statement. Similarly in lines 150-152.

Indeed, Ref [25] is the article on the “Amber 99SB* ILDN-DERK” force field, not related to the overstablized interactions [24]. We thus removed reference [25] from the two places mentioned above.

- The authors suggest that salt bridges are overstabilized based on differences in Rg (p. 8, line 150-152). Rg is an global feature. This statement could be supported quantitatively by providing populations of salt bridges in the simulations with differing salt concentrations.

This is an important point. We calculated the salt bridges for Ash1/pAsh1 (for CTD2’, there are not any positively charged residues in the sequence, thus salt bridges do not exist) under different NaCl concentrations. The results, shown in Fig. S2, are in line with our conclusion: 300-400 NaCl yields the best agreement with experiments and also results in a smaller average number of salt bridges than lower salt concentrations. Further increasing salt does not reduce salt bridges any further.

We discussed from line 175 of page 9 as below:

“Indeed, we observe fewer salt bridges within pAsh1 for the range of 300-400mM NaCl compared to lower salt concentrations, suggesting that higher salt indeed reduces the overstabilization of salt bridges inherent to the force field (Fig. S2).”

- When comparing to SAXS/NMR measurements, it would be useful to provide the salt concentrations in these measurements.

The reverted calculated SAXS curve from MD simulations are under 350 mM NaCl. As Waxsis requires a huge simulation box, we did not extend this analysis to other concentrations. We have added “350 mM NaCl” to Figure 3. For the NMR, we now calculated the chemical shifts for other concentrations in Fig. S3.

The SAXS and NMR experiment for Ash1/pAsh1 are both performed under 150 mM NaCl, we have also added the information in Fig. 3 and 4.

- On p. 9, the authors state that “We conclude that using 350mM NaCl yields conformational ensembles of the two IDPs and their phosphorylated forms which agree with SAXS and NMR data and thus resemble the experimental ensemble.” Is 350mM the concentration for which the agreement is best? Do the SAXS and NMR data both show the best agreement at the same concentration?

Actually, we do not want to claim that 350mM is the best choice but instead, in line of the uncertainties in both experiments and simulations, emphasize that a higher salt concentration in the range of 300-400mM is a valid choice. Accordingly, in line 172 of page 9, we conclude that “more generally concentrations in the 300-400mM range” best mimic the experimentally observed behavior of Ash1/pAsh1 and CTD2’/pCTD2’.

We now calculated chemical shifts across the simulated salt concentrations and find them to be largely insensitive to the salt concentration, likely because chemical shifts measure the local environment and salt primarily alters the global propensities. However, the RMSD from experimental chemical shifts for hydrogens are smaller in the range of 300-400mM compared to the lower range, and also are similar on average across the atom types.

We now discuss this comparison in further detail on page 9 line 181.

“The overall RMSDs from experimental chemical shift values are largely robust with regard to salt concentrations, with a slight decrease in RMSD for hydrogen chemical shifts in the range of 300-400mM compared to lower salt, corroborating that higher salt improves the overall quality of the predicted Ash1/pAsh1 ensembles (Table S1 and Fig. S3).”

- What method was used to compute the chemical shifts? Can the authors provide error estimates for these shifts in Fig. 2? They state that the agreement is “very good” but without reporting error or the method used to compute the shifts, it is hard to assess this. Which salt concentration was used in the simulations reported in Fig. 2?

We used shiftx2 to calculate chemical shifts, we now mention this in Methods (page 25). We now provide the RMSD for all salt concentrations in Table S1 (see point above). We have added this information in the methods part. We now also specify the salt concentration in Fig. 3 (previously Fig. 2).

- p. 11, lines 191-193: The authors’ assessment of the accuracy of the C36m force field is problematic. They have carried out only a single simulation of 180 ns for 4 systems (~30 times less simulation than other systems). Despite the short simulation, they claim “In the case of Charmm36m, irrespective of the IDP and the phosphorylation, we observe a strong over collapse of the IDP which is not in line with the SAXS data and dominate over the charge effect.” This statement isn’t supported by the data.

We have carried out further MD simulations with the Charmm36m force field for Ash1/pAsh1 and CTD2’/pCTD2’ under 100 mM NaCl, namely 7 trajectories with different initial structure and 400 ns for each trajectory. The conformations are largely over-collapsed, confirming our previous conclusion. The results are shown in Fig. S6.

In line 203 of page 10, we discussed as below:

“In the case of Charmm36m, irrespective of the IDP and the phosphorylation, we observe a strong overcollapse of the IDP which is not in line with the SAXS data (Fig. S6).”

- The paper would benefit from a systematic analysis of salt bridges, rather than relying on Rg (a global structural property) to infer effects on local structure, like salt bridges.

As a response to a previous comment, we have analyzed the change in salt bridges when modifying the NaCl concentration, see above and Fig. S2.

In addition, we now have calculated the salt bridges for Ash1/pAsh1 and E-Cadherin/pE-Cadherin and in the revised version compare them in new Figure 6.

We discuss the findings from line 272 of page 13:

“We analyzed the salt bridge propensities and found salt bridges involving the phosphate groups, but not the other salt bridges, to strongly correlate with the overall shrinking and swelling behavior described above (Fig. 6). For example, the positively charged IDP Ash1 shrinks upon phosphorylation, involving - and likely driven by - a significant increase of salt bridges (Fig. 6A). Instead, salt bridges of negatively charged E-Cadherin only increase slightly upon phosphorylation (Fig. 6B), as a strong repulsive force from negatively charged residue oppose their formation in pE-Cadherin. We observed an only limited influence of IDP phosphorylation on the number of D/E – R/K salt bridges for Ash1 (Fig. 6C), suggesting direct phosphate-D/E/R/K interactions to drive the observed changes in IDP dimensions in the case of positively charged IDPs (Fig. 6E). When it comes to E-Cadherin, D/E-R/K interactions were strongly reduced upon phosphorylation because of the expansion of the conformations (Fig. 6D), and only partly compensated by newly established salt bridges involving phosphates (Fig. 6F). Salt bridges thus play very important roles in shaping the conformations of IDPs (Fig. 6G).”

- p. 14, line 234: The authors refer to “the hydrodynamic radius”, but no RH is reported, so I think this must be a typo.

Rg from SAXS includes the contribution from ions and solvent, while the Rg from MD simulations only measures the mass distribution of the protein itself.

We have clarified this and now always refer to the “radius of gyration”:

“The simulation-derived SAXS data takes the solvation shell into account, thus measuring not only the protein by itself but the radius of gyration with contributions from ions and solvent, in direct analogy to the experiments [18,34].” from line 227 of page 11.

- p. 15, line 259: The authors suggest that the plot shown in Fig. 4 has a V-shape, but given the large error, this isn’t well-supported by the results.

To quantify the significance of the V-shape for the two different salt concentrations, we mirrored the left part of the V-shape, and conducted a linear fitting (considering the errors in Rg) over the 4 datasets, for both the 100 mM and 350 mM simulations independently. We obtained p-values smaller than 0.05 for both salt concentrations. This analysis is shown in new Fig. S9.

We discuss this from line 261 of page 13:

“The correlation of the normalized RG with the absolute value of NCPR is significant, with a slope from linear fits significantly different from zero (p-values <0.05, Fig. S9).”

- Fig. 5 is unclear and difficult to interpret.

We now use box plots plus a simple scheme to better summarize the results. See new Fig. 6 for more details.

- It is unclear how the equilibration of the simulations was treated. E.g. in Fig. S4, the authors exclude 40 ns for equilibration, but the reason for this decision wasn’t clear. Was 40 ns used for all systems? If so, why?

We had rerun the charmm36m simulations and use the same criteria as other force fields. The simulations are performed for 400 ns for each trajectory, while the first 100 ns were not used in analyses, which is the same for production runs using other force fields. We clarify this in the Methods section.

- Have the authors compared to all available experimental data for these systems?

Yes. For Ash1, we have compared the SAXS and NMR chemical shifts, both were made available to us. For CTD2’, only SAXS data was compared, as chemical shifts are not available to us. There is no experimental data available for E-Cadherin and p130cas.

**Have all data underlying the figures and results presented in the manuscript been provided?**
Large-scale datasets should be made available via a public repository as described in the *PLOS Computational Biology* [data availability policy](http://journals.plos.org/ploscompbiol/s/data-availability), and numerical data that underlies graphs or summary statistics should be provided in spreadsheet form as supporting information.

Reviewer #1: Yes

Reviewer #2: **No:** Numerical data for the figures has not been provided.

We have provided all the numerical data for figures in Data S1.
